# *In vitro* and *in vivo* osteogenesis of rat adipose-derived stem cells combined with calcium alginate gel scaffold induced by calcitonin gene-related peptide

**DOI:** 10.3389/fcell.2025.1669459

**Published:** 2025-09-12

**Authors:** Changzhi Huang, Xiaofeng Liu, Liang Lin, Shimin Zhang, Nanyi Xu, Xiaoyong Wang, Jiuzao Lin

**Affiliations:** ^1^ Department of Joint Surgery and Sports Medicine, Ningde Municipal Hospital of Ningde Normal University, Ningde, Fujian, China; ^2^ Ningde Clinical Medical College of Fujian Medical University, Ningde, Fujian, China; ^3^ Department of Orthopaedics, Shanghai Sixth People’s Hospital Fujian, Quanzhou, Fujian, China; ^4^ Clinical Research Center for Orthopaedic Trauma and Reconstruction of Fujian Province, Quanzhou, Fujian, China; ^5^ Department of Orthopaedics, The First Hospital of Putian City, Putian, Fujian, China; ^6^ The School of Clinical Medicine, Fujian Medical University, Fuzhou, China

**Keywords:** adipose-derived stem cells, calcitonin gene-related peptide, calcium alginate hydrogel, tissue engineering, three-dimensional culture, osteogenic differentiation

## Abstract

**Backgroud:**

Bone defect repair is clinically challenging due to the limitations of traditional treatments. Tissue engineering holds great potential for constructing bone substitutes. This study evaluates the osteogenic capability of calcitonin gene-related peptide (CGRP)-induced rat adipose-derived stem cells (ADSCs) combined with calcium alginate (CaAlg) scaffolds both *in vitro* and *in vivo*.

**Methods:**

ADSCs were isolated from rat inguinal fat pads, cultured, and characterized at passage 3. For *in vitro* experiments, cells were grouped and assessed over time using the CCK-8 assay for proliferation, alkaline phosphatase (ALP) activity assays, ALP staining, alizarin red staining (ARS), RT-PCR, and Western blotting for osteogenesis-related gene and protein expression. For *in vivo* experiments, constructs were evaluated after 12 weeks using X-ray, micro-CT, gross observation, and H&E staining.

**Results:**

ADSCs had clear surface antigen characteristics and displayed an “S”-shaped proliferation curve post-osteogenic induction. *In vitro*, CGRP and CaAlg scaffolds synergistically enhanced ADSC osteogenic differentiation, with higher early ALP activity and late-stage mineralization in the CGRP-ADSCs-CaAlg group. Additionally, osteogenesis-related gene and protein expressions were upregulated in CGRP-induced and scaffold-combined groups. *In vivo*, bone formation was observed in both ADSCs-CaAlg and CGRP-ADSCs-CaAlg groups, but not in the control group.

**Conclusion:**

These findings indicate that CGRP can induce ADSCs combined with CaAlg scaffolds to form tissue-engineered bone *in vivo*, with CGRP and CaAlg scaffolds showing a synergistic effect on promoting ADSC osteogenic differentiation.

## 1 Introduction

Bone defects, resulting from trauma, infection, tumor resection, or corrective surgery, represent a common clinical challenge in orthopedics (Seebach et al., 2024; Monje et al., 2023; Aldhaher et al., 2023). When defects exceed a critical size, they typically fail to heal spontaneously and require surgical intervention (Arpitha et al., 2023; Hopkins et al., 2023). Common treatment options include autologous bone grafts, allogeneic bone transplantation, and synthetic bone substitutes (Tournier et al., 2021; Diachkova et al., 2022). Although autografts and allografts can effectively promote bone regeneration, they are associated with significant limitations. Autografts are limited in availability and may lead to donor-site morbidity, such as pain, infection, or secondary injury. Allografts and synthetic materials, while avoiding donor-site complications, carry risks of immune rejection, disease transmission, and poor integration (Migliorini et al., 2021; Misch, 2022; Schmidt, 2021; Li et al., 2023; Xie et al., 2021). These constraints have motivated the search for alternative bone repair strategies, particularly in the field of bone tissue engineering (BTE). Bone tissue engineering typically combines three key elements: bioactive factors, seed cells, and scaffold materials. This approach aims to enhance bone regeneration by facilitating the osteogenic differentiation of seed cells through the use of bioactive molecules and biomaterial scaffolds (Koushik et al., 2023; Heng et al., 2023).

Calcitonin gene-related peptide (CGRP) is a neuropeptide widely distributed in the peripheral and central nervous systems. Growing evidence indicates that CGRP can promote osteogenic differentiation in various stem cell types—including adipose-derived stem cells (ADSCs), bone marrow mesenchymal stem cells, periodontal ligament stem cells, and dental pulp stem cells—when combined with biomaterial scaffolds (Jia et al., 2019; Lv et al., 2022). ADSCs have emerged as a promising cell source for tissue engineering due to their abundant supply, accessibility, high proliferative capacity, and multilineage differentiation potential. They can differentiate into adipogenic, osteogenic, chondrogenic, myogenic, endothelial, and neural lineages, among others (Liu et al., 2007; Lotfy et al., 2014; Fraser et al., 2006). Calcium alginate (CaAlg) hydrogel is a biocompatible, biodegradable, and non-toxic scaffold material with low immunogenicity. It has been widely applied as an injectable carrier in bone and cartilage tissue engineering, demonstrating excellent support for ADSC viability and function (Fang et al., 2013).

Although studies have demonstrated the osteogenic-promoting effects of CGRP, most research has focused on bone marrow mesenchymal stem cells (BMSCs) (Jia et al., 2019; Li et al., 2021; Guo et al., 2020; Zhang et al., 2019), with relatively limited investigation into ADSCs (Fang et al., 2013). Given that ADSCs possess similar multilineage differentiation potential (Zuk et al., 2001), gene expression profiles, and osteogenic capacity as BMSCs (Ding et al., 2010), while also exhibiting higher yield and proliferation ability than BMSCs (Liao et al., 2010; Tsuji et al., 2014; Li et al., 2016), ADSCs have emerged as a promising alternative seed cell type and are expected to become a focus in bone and cartilage tissue engineering research. Previous studies have indicated that CGRP can induce and promote the osteogenic differentiation of ADSCs (Fang et al., 2013; Huang et al., 2015). Based on this foundation, the present study innovatively combines ADSCs loaded in sodium alginate hydrogel with CGRP-induced osteogenic differentiation to construct tissue-engineered bone, thereby providing a tissue engineering strategy for bone defect repair. This approach helps overcome limitations associated with BMSCs, such as difficult harvesting and limited availability.

Although our previous *in vitro* study (Huang et al., 2015) showed that CGRP promotes the osteogenic differentiation of ADSCs within a three-dimensional (3D) CaAlg hydrogel environment, its *in vivo* osteogenic potential remains unclear. To our knowledge, no similar studies have been reported. Therefore, this study aims to evaluate whether ADSCs encapsulated in an injectable CaAlg hydrogel system can enhance bone formation under CGRP induction, offering a feasible therapeutic strategy for bone defect repair. We hypothesize that the combination of ADSCs, CGRP, and CaAlg hydrogel can synergistically promote engineered bone formation.

## 2 Materials and methods

### 2.1 Ethical statement

All experimental protocols were approved by the Institutional Animal Care and Use Committee of Fujian Medical University (Approval Number: IACUC-FJMU-20220804) and conducted in accordance with the Guidelines for the Care and Use of Laboratory Animals.

### 2.2 Study design

This study included the preparation of CaAlg hydrogel, *in vitro* cellular experiments, and *in vivo* animal experiments. Data were analyzed using one-way analysis of variance (ANOVA).

### 2.3 Time and location

The experiments were conducted from July 2024 to April 2025 at the Fujian Orthopaedic Research Institute, the First Affiliated Hospital, Fujian Medical University.

### 2.4 Materials

#### 2.4.1 Experimental animals

A total of 20 male SPF-grade Sprague-Dawley (SD) rats, aged 8 weeks and weighing 200–300 g, were provided by SibeiFu (Suzhou) Biotechnology Co., Ltd. (Production License No. SCXK [Su] 2022-0006; Use License No. SYXK [Min] 2025-0003). The animals were housed in the Animal Center of Fujian Orthopaedic Research Institute, acclimatized for 7 days under standard conditions: temperature 20 °C – 25 °C humidity 45%–50%, and a 12-h light/dark cycle, with free access to food and water. All experimental procedures complied with ethical guidelines for animal research and were approved by the Institutional Animal Care and Use Committee.

#### 2.4.2 Main reagents and instruments

Cell proliferation assay kit, BCA protein concentration assay kit (Beyotime Biotechnology, Shanghai, China); BCIP/NBT alkaline phosphatase (ALP) staining kit, alizarin red staining (ARS) solution, alkaline phosphataseactivity assay kit (Beijing Yita Biotechnology, Beijing, China); hematoxylin staining solution, eosin staining solution, paraformaldehyde solution (Cida Biotechnology, Guangzhou, China); osteocalcin (OCN) and runt-related transcription factor 2 (RUNX2) rabbit monoclonal antibodies (Abcam, USA); RNA extraction kit, RT kit, PCR reaction mixture (TianGen, Beijing, China); PCR primers (Shanghai Sangon Biotech, Shanghai, China); CO_2_incubator (Thermo Fisher Scientific, USA); flow cytometer (Beckman Coulter, USA); microplate reader, spectrophotometer (BioTek, USA); inverted microscope (BD Biosciences, USA); PCR amplifier (MJ Research, USA); UV gel imaging system (Alpha Innotech, USA); Western blot electrophoresis system (Bio-Rad, USA); single-slice CT scanner (Siemens, Germany).

### 2.5 Methods

#### 2.5.1 Cell isolation and culture

Six-week-old healthy male SD rats were anesthetized with 0.2% pentobarbital sodium, and bilateral inguinal adipose tissues were harvested and minced into fine fragments. The tissue fragments were digested with 0.1% type I collagenase, and the digestion was terminated by adding DMEM/F-12 basal medium. The resulting cell suspension was filtered through a 200-mesh nylon mesh and centrifuged at 1,200 rpm for 5 min. After discarding the supernatant, the cell pellet was resuspended in DMEM/F-12 medium supplemented with 10% fetal bovine serum (FBS) (Gibco, Invitrogen, US) and 1% double antibiotics (Gibco, Invitrogen, US), adjusted to a density of 1 × 10^6^ to 1 × 10^7^ cells/mL, and seeded into 25 cm^2^ (T25) culture flasks. The flasks were incubated in a humidified atmosphere of 5% CO_2_ at 37 °C. The culture medium was first changed after 24 h to remove non-adherent cells, and subsequently replaced every 2–3 days. Cell morphology and growth were monitored under an inverted phase-contrast microscope. When the cells reached 80%–90% confluence, they were digested with 0.25% trypsin-EDTA and subcultured at 1:3 ratio. Third-passage ADSCs was used for all subsequent experimental procedures.

#### 2.5.2 ADSCs characterization

Third-passage cells were collected, digested with 0.25% trypsin-EDTA, and centrifuged at 1,200 rpm for 5 min. The supernatant was discarded, and the cell pellets were resuspended and aliquoted into EP tubes. FITC- and PE-conjugated antibodies against CD29, CD31, CD44, CD45, and CD105 were added to the respective tubes. FITC-labeled goat IgG and PE-labeled mouse IgG were used as isotype controls. The cells were incubated in the dark for 20–30 min, and then analyzed by flow cytometry to determine the expression of surface markers.

#### 2.5.3 Preparation of the hydrogel and experimental grouping

Third-passage ADSCs were adjusted to a cell density of 1 × 10^8^ cells/L and mixed with 2 mL of 0.5% sodium alginate solution containing CGRP. The mixture was thoroughly pipetted to ensure uniformity, and 50 µL droplets, each containing approximately 2,500 cells, were dispensed into a 24-well plate pre-filled with 1 mL of 2% CaCl_2_ per well. Each well received a total volume of 200 µL of the cell-alginate mixture, and the experiment was conducted in 12 replicates per group. The hydrogel was allowed to form at room temperature and subsequently used for further experimental analysis. In parallel, ADSCs-laden calcium alginate hydrogels without CGRP were prepared using the same method. For osteogenic induction, the CGRP-ADSCs-CaAlg group was cultured in osteogenic medium supplemented with 1.5 µg/L CGRP, whereas the ADSCs-CaAlg group was maintained in standard osteogenic medium containing 10 mmol/L β-glycerophosphate, 50 mg/L ascorbic acid, and 10 nmol/L dexamethasone. Meanwhile, third-passage ADSCs were seeded at a density of 1 × 10^6^ cells/mL onto coverslips in 6-well plates and divided into two groups: the ADSCs group and the CGRP-ADSCs group. The preparation of ADSCs-loaded hydrogel for animal experiments followed the same procedure as described above.

#### 2.5.4 Observation of ADSCs growth morphology in CaAlg hydrogel scaffolds

Following the incorporation of ADSCs into calcium alginate hydrogel scaffolds, the growth and morphology of the cells within the scaffolds were monitored using an inverted phase-contrast microscope. Observations were conducted at specific time points: 1, 3, 5, 7, and 9 days after the initial combination of ADSCs with the hydrogel. This allowed for the assessment of cell attachment, spreading, proliferation, and overall morphological changes over time within the three-dimensional scaffold environment.

#### 2.5.5 Cell proliferation assay using CCK-8 after osteogenic induction

To assess the proliferation activity of ADSCs after osteogenic induction, cells were collected at 1, 3, 5, 7, and 9 days post-induction. For each time point, six replicates per group were used. The composite carriers were first dissolved using 55 mmol/L sodium citrate, and the cells were centrifuged and resuspended in 96-well plates. Each well received 90 µL of DMEM/F-12 medium containing 10% FBS and 1% penicillin-streptomycin, followed by the addition of 10 µL of CCK-8 solution. The plates were incubated at 37 °C in the dark for 4 h. Absorbance (optical density, OD) values were measured at a wavelength of 450 nm using a microplate reader. The mean OD value from six wells was plotted on the y-axis, with time on the x-axis to generate a standard curve.

#### 2.5.6 ALP staining and activity assay

At 7 and 21 days post-osteogenic induction, cells from each group were collected (four replicates per group). The composite carriers were first dissolved using 55 mmol/L sodium citrate, and the cells were centrifuged and resuspended at a density of 5 × 10^4^ cells per well in 96-well plates. After 24 h, the cells were fixed with 4% paraformaldehyde and incubated with 200 µL of BCIP/NBT alkaline phosphatase staining solution per well in the dark for 1 h. Images were captured following staining. After staining, the cells were lysed with RIPA lysis buffer. The lysate was centrifuged at 8,000 rpm for 3 min, and the supernatant was used to measure both ALP activity and total protein concentration using a BCA assay. ALP activity was normalized to total protein content to obtain quantitative results.

Additionally, at 3, 7, 14, and 21 days post-induction, cells were collected from each group (four replicates per group), and the composite carriers were again dissolved with 55 mmol/L sodium citrate. Following centrifugation, the cells were transferred to a 96-well plate and treated with 100 µL of 1% Triton X-100 and incubated overnight at 4 °C. ALP activity was then measured according to the manufacturer’s instructions for the ALP assay kit. ALP activity was expressed as units per liter (U/L).

#### 2.5.7 Alizarin red staining

At 7 and 21 days post-osteogenic induction, cells from each group were collected and seeded into 96-well plates at a density of 5 × 10^4^ cells per well. The cells were fixed with 4% paraformaldehyde for 30 min, rinsed with PBS, and then incubated with 200 µL of 2% alizarin red staining solution at room temperature for 20 min. The formation of mineralized nodules was observed and photographed under an inverted phase-contrast microscope. After staining, 1% cetylpyridinium chloride (CPC) solution was added to each well to extract the bound alizarin red. The absorbance was measured at 562 nm using a spectrophotometer to quantify the staining intensity, which reflects the extent of calcium deposition.

#### 2.5.8 RT-PCR detection of RUNX2 and OCN mRNA expression

At 7 and 14 days post-osteogenic induction, the cultures were terminated. The composite carriers were dissolved using 55 mmol/L sodium citrate, and the cells were collected by centrifugation. Total RNA was extracted using Trizol reagent, and RNA purity was assessed before reverse transcription was performed with an RT kit to synthesize cDNA. PCR amplification was then carried out using specific primers for RUNX2 (forward: 5′-TCAACGATCTGAGATTTGTGGG-3′, reverse: 5′-GGGGAGGATTTGTGAAGACGG-3′) and OCN (forward: 5′-AGGGCAGTAAGGTGGTGAA-3′, reverse: 5′-GTCCTGGAAGCCAATGTGGTCA-3′). The PCR reaction began with an initial denaturation at 95 °C for 1 min, followed by 30 cycles of denaturation at 95 °C for 10 s, annealing at 56 °C for 20 s, and extension at 75 °C for 1 min, with a final extension at 75 °C for 10 min. The resulting PCR products were analyzed by agarose gel electrophoresis and DNA absorbance scanning. mRNA expression levels of RUNX2 and OCN were semi-quantified by comparing the band intensities of the target genes with that of the internal control β-actin.

#### 2.5.9 Western blot detection of RUNX2 and OCN protein expression

At 7 and 14 days post-osteogenic induction, the cultures were terminated. The composite carriers were dissolved using 55 mmol/L sodium citrate, and the cells were collected by centrifugation. Cells were lysed using RIPA lysis buffer to extract total protein. Protein concentrations were determined using the Bradford assay. Equal amounts of protein were separated by sodium dodecyl sulfate-polyacrylamide gel electrophoresis (SDS-PAGE) and then transferred onto polyvinylidene difluoride (PVDF) membranes. The membranes were blocked with 5% bovine serum albumin (BSA) in Tris-buffered saline with Tween 20 (TBST) for 1 h at room temperature. Membranes were subsequently incubated with primary antibodies against OCN, RUNX2, and β-actin (diluted 1:1,000) overnight at 4 °C. On the second day, the membranes were washed with TBST and incubated with secondary antibodies for 1 h at room temperature. Protein bands were visualized using enhanced chemiluminescence (ECL) detection reagents, followed by exposure and development to quantify the expression levels of the target proteins.

#### 2.5.10 *In Vivo* osteogenesis of ADSCs combined with calcium alginate gel

Eighteen healthy adult male SD rats weighing 180–200 g were used in this study. The animals were anesthetized with 0.2% pentobarbital sodium and underwent bilateral dorsal depilation using 8% sodium sulfide. After fixation, disinfection, and draping, three longitudinal incisions were made on each rat’s back. One muscle pouch was created on the left side and two on the right side by incising the skin and subcutaneous tissue and separating the underlying muscles to form implantation sites. The pouches were rinsed with sterile saline, and each animal received one type of graft randomly implanted into its three muscle pouches: the left pouch was implanted with CGRP-ADSCs-CaAlg, the upper right pouch with CaAlg alone (control group), and the lower right pouch with ADSCs-CaAlg. The skin was then sutured. Postoperatively, wound care was performed daily, and the animals were housed individually. To prevent infection, gentamicin (80,000 units) was administered intramuscularly once daily for three consecutive days. During the first postoperative week, surgical sites were monitored daily, followed by weekly observations thereafter. At 12 weeks post-implantation, radiographic imaging (X-ray) and micro-CT scanning were performed prior to euthanasia. The implants were then harvested for macroscopic evaluation of bone formation, followed by histological analysis using hematoxylin and eosin (H&E) staining to assess *in vivo* osteogenic potential.

### 2.6 Main observation indicators

The primary observation indicators included the assessment of osteogenic markers through *in vitro* cell staining, as well as the evaluation of osteogenesis-related gene and protein expression using RT-PCR and Western blot techniques. Specifically, ALP staining and alizarin red staining were employed to identify early and late osteogenic differentiation markers, respectively. The mRNA expression levels of key osteogenic genes such as RUNX2 and OCN were quantified by RT-PCR, while their corresponding protein expressions were detected via Western blot analysis. For *in vivo* studies, gross visual inspection, radiographic imaging (X-ray), and micro-CT scanning were performed to evaluate bone formation macroscopically. Additionally, histological examination using H&E staining was conducted to assess osteogenesis at the tissue level.

### 2.7 Statistical analysis

All experimental data were analyzed using SPSS 26.0 software (IBM, Armonk, New York, USA). Data are expressed as mean ± standard deviation (Mean ± SD). Comparisons among multiple groups were performed using one-way analysis of variance (ANOVA), followed by Tukey’s *post hoc* test for multiple pairwise comparisons. A P value less than 0.05 was considered statistically significant. The statistical methods used in this study have been reviewed and approved by a biostatistics expert from Ningde Normal University.

## 3 Results

### 3.1 Morphological observation of ADSCs growth

**FIGURE 1:**
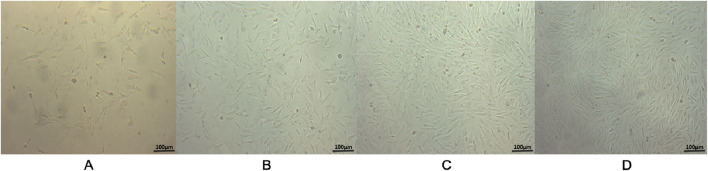
Observation of ADSCs Growth Morphology (×100). **(A)** Primary cells after the first medium change, with non-adherent cells removed, showed most adherent cells exhibiting a short spindle or polygonal shape. **(B)** After 3 days of culture, primary cells reached approximately 50% confluence and displayed an elongated spindle morphology. **(C)** By 6 days of culture, primary cells reached about 90% confluence, exhibiting typical elongated spindle shapes arranged in a “vortex-like” pattern. **(D)** Third-passage cells cultured for 3 days maintained the typical elongated spindle morphology and vortex-like arrangement. Scale bar = 100 µm.

The morphology of ADSCs was observed during *in vitro* culture. Initially, the isolated cells appeared round and began to adhere within 24–48 h after seeding. Non-adherent cells, primarily red blood cells, were removed during the first medium change. At this stage, most of the adherent cells exhibited a short spindle or polygonal shape (Figure 1A). As culture time progressed, the cell number increased and the morphology became more elongated and fibroblast-like. The cells grew in clusters and exhibited a colony-forming tendency (Figure 1B). By day 5–7, the cells reached approximately 80%–90% confluence and arranged in a “vortex-like” pattern (Figure 1C). Following passage, the cells displayed an accelerated growth rate, reaching confluence again in about 3–4 days, and could be stably cultured over long-term passages (Figure 1D).

### 3.2 Flow cytometry characterization of ADSCs

**FIGURE 2:**
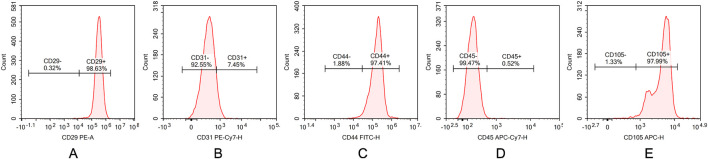
Characterization of ADSCs by flow cytometry. **(A)** CD29, **(B)** CD31, **(C)** CD44, **(D)** CD45, and **(E)** CD105 surface marker expressions in ADSCs. Flow cytometric analysis demonstrating high expression of mesenchymal markers CD29, CD44 and CD90 (98.63%, 97.41%, and 97.99%) with minimal expression of hematopoietic marker CD31,CD45 (7.45%, 0.52%).

Flow cytometry was used to analyze the surface antigen expression of third-passage ADSCs. The results demonstrated that the cells were positive for CD29, CD44, and CD105, with positive rates of 98.63%, 97.41%, and 97.99%, respectively. Conversely, the cells were negative for CD31 and CD45, with negative rates of 92.55% and 99.47%, respectively (Figure 2). These findings are consistent with the known surface antigen expression profile of ADSCs, confirming that the cells isolated using the collagenase digestion and adherent culture method, and subsequently passaged, are indeed ADSCs.

### 3.3 Morphological characteristics of ADSCs in calcium alginate gel scaffolds

**FIGURE 3:**
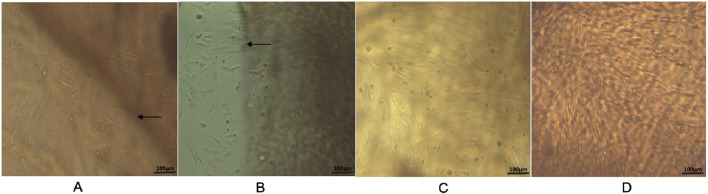
Inverted microscope observation on the composite of ADSCs and calcium alginate gel scaffold (×100). **(A)** Cells cultured on calcium alginate hydrogelscaffolds for 24 h; **(B)** cells cultured for 3 days; **(C)** cells cultured for 7 days; and **(D)** cells cultured for 9 days. Microscopy showing typical spindle-shaped morphology of third-passage ADSCs cultured on calcium alginate hydrogel scaffolds at different time points. Scale bar = 100 µm. Black arrows indicate the boundary of the calcium alginate hydrogel scaffold.

At 24 h post-seeding, ADSCs adhered to the surface of the calcium alginate gel scaffolds. Some cells aggregated into clusters, displaying varied morphologies including round and short spindle shapes. The cell density was low, resulting in large gaps between cells (Figure 3A). By day 3, the gaps between cells decreased, with most cells adopting an elongated spindle shape, although a small portion remained round (Figure 3B). At day 7, the cells had fused into a confluent layer covering the entire surface of the calcium alginate gel scaffold. They arranged in a characteristic “vortex-like” pattern, maintaining their elongated spindle morphology and forming continuous sheets with neighboring cells (Figure 3C). By day 9, the cells continued to form a confluent monolayer without significant changes in growth status or morphology (Figure 3D).

### 3.4 CCK-8 assay for the proliferation activity of ADSCs after osteogenic induction

**FIGURE 4:**
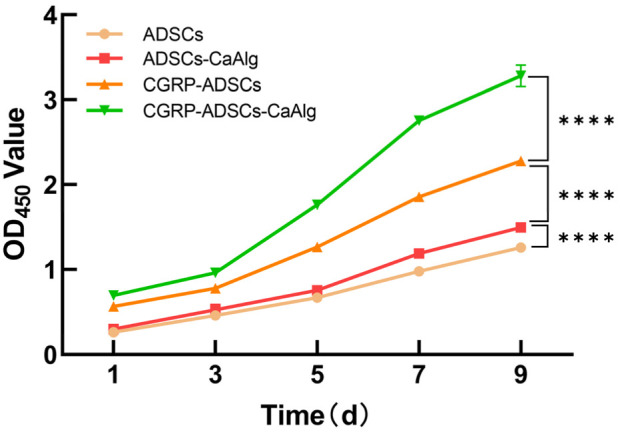
Comparison of proliferation activity of ADSCs after osteogenic induction. The proliferation activity of cells in each group was assessed using the CCK-8 assay. OD values were measured at 450 nm at different time points, and growthcurves were plotted accordingly. All groups of ADSCs showed robust proliferation and differentiation, with the logarithmic growth phase occurring between days 3–7, followed by entry into a plateau phase by day 9. Statistical analysis revealed significant differences in cell proliferation activity among the groups (****P < 0.0001).

The proliferation activity of ADSCs following osteogenic induction was assessed using the CCK-8 assay on days 1, 3, 5, 7, and 9. A growth curve was constructed with time as the x-axis and the mean OD values from six wells as the y-axis (Figure 4). The results demonstrated that all groups of ADSCs showed robust proliferation and differentiation, with the logarithmic growth phase occurring between days 3–7, followed by entry into a plateau phase by day 9. Significant differences were observed among the groups at each time point (*P* < 0.0001, Figure 4). Specifically, ADSCs cultured either in the presence of CaAlg scaffolds or CGRP exhibited significantly enhanced growth and differentiation compared to the ADSCs-only group (*P* < 0.001), while CGRP exerted a more pronounced effect on ADSC proliferation and differentiation than CaAlg alone (*P* < 0.001). Notably, the combination of CGRP with ADSCs and CaAlg (CGRP-ADSCs-CaAlg) resulted in the most rapid proliferation and differentiation rates (*P* < 0.001). These findings collectively indicate that both CGRP and CaAlg promote ADSC proliferation and differentiation, and their combined application yields a synergistic enhancement effect.

### 3.5 ALP staining and activity assay

**FIGURE 5:**
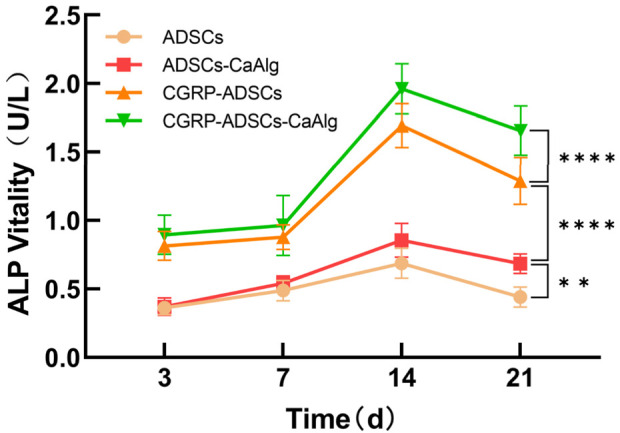
Trend of ALP activity changes after osteogenic induction. The expression of the osteogenic differentiation marker ALP was measured at different time points for each group. The CGRP-treated groups consistently exhibited higher ALP concentrations compared to the non-CGRP-treated groups referred to as the basal induction groups) at all time points, and the differences were statistically significant. Allgroups reached peak ALP levels at approximately 2 weeks post-induction, followed by a gradual decline there after. Statistical analysis revealed significant differences in ALP activity among the groups (**P < 0.01; ****P < 0.0001).

ALP activity was measured on days 3, 7, 14, and 21 after osteogenic induction, while ALP staining was performed on days 7 and 21. As shown in Figure 5, there were significant inter-group differences in ALP levels. The CGRP-treated groups (CGRP-ADSCs and CGRP-ADSCs-CaAlg) consistently exhibited higher ALP concentrations compared to the non-CGRP-treated groups (ADSCs and ADSCs-CaAlg, referred to as the basal induction groups) at all time points, and the differences were statistically significant (*P* < 0.05). The ALP concentration profiles also varied among the groups, with greater fluctuations observed in the CGRP-treated groups. All groups reached peak ALP levels at approximately 2 weeks post-induction, followed by a gradual decline there after. An ALP concentration curve was plotted using the mean ALP values from six wells as the y-axis and time as the x-axis (Figure 5).

**FIGURE 6:**
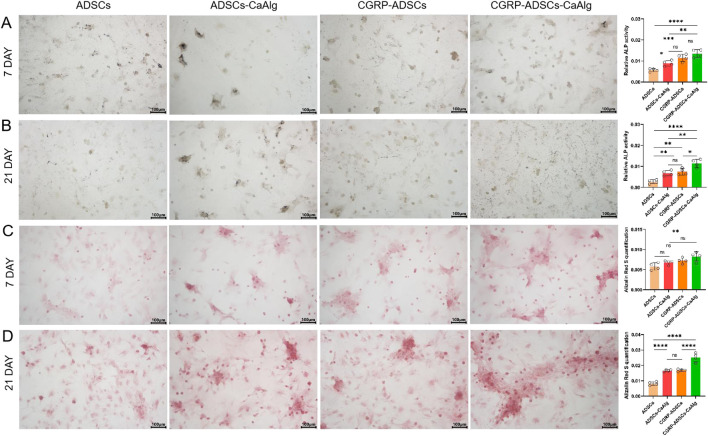
Osteogenesis on various samples *in vitro*. **(A,B)** ALP staining and quantitative analysis of ADSCs in osteogenic induction culture among all groups after 7 days and 21 days. ALP expression was most prominent on day 7 and decreasedby day 21, indicating that ALP serves as an early marker of osteogenic differentiation. CGRP-treated groups showed significantly higher ALP expression on day 7 compared to the basal induction groups suggesting that CGRP enhances the osteogenic differentiation of ADSCs. scale bar: 100 μm. Data are presented as mean ± SD (n = 4 biologically independent experiments). *P*-values were calculated using one-way ANOVA with Tukey’s *post hoc* test: ns means not significant. *P < 0.05, **P < 0.01, ***P < 0.001 and ****P < 0.0001. **(C,D)** Alizarin red staining for calcium mineralization and quantitative analysis of ADSCs in osteogenic induction culture among all groups after 7 days and 21 days. The number of calcium nodules was relativelylow at day 7 but significantly increased by day 21, where the nodules appeared large, round, and coalesced into sheet-like formations with clear layers. CGRP-treated groups showed a significantly higher number of calcium nodules at day 21 compared to the non-CGRP-treated groups, with statistical significance. scale bar: 100 μm.Data are presented as mean ± SD (n = 4 biologically independent experiments). *P*-values were calculated using one-way ANOVA with Tukey’s *post hoc* test: ns means not significant. *P < 0.05, **P < 0.01, ***P < 0.001 and ****P < 0.0001.

On days 7 and 21 of osteogenic induction, ALP staining was positive in all experimental groups. Under inverted phase-contrast microscopy, numerous cells displayed deep blue nuclei and blue-black cytoplasmic staining (Figure 6). ALP expression was most prominent on day 7 and decreased by day 21, indicating that ALP serves as an early marker of osteogenic differentiation. Notably, the CGRP-treated groups showed significantly higher ALP expression on day 7 compared to the basal induction groups (0.0115 ± 0.0017 vs. 0.0056 ± 0.0008 and 0.0135 ± 0.0019 vs. 0.0089 ± 0.0013, both *P* < 0.05), suggesting that CGRP enhances the osteogenic differentiation of ADSCs. Among all four groups, the CGRP-ADSCs-CaAlg group exhibited the strongest ALP expression, demonstrating that CGRP and calcium alginate hydrogel exert a synergistic effect in promoting osteogenic differentiation of ADSCs.

### 3.6 Alizarin red staining for osteogenic differentiation

Alizarin Red staining (ARS) was performed on days 7 and 21 after osteogenic induction to detect mineralized nodules. Under inverted phase-contrast microscopy, numerous oval-shaped calcium nodules stained in orange-red were observed in all groups (Figure 6). The number of calcium nodules was relatively low at day 7 but significantly increased by day 21, where the nodules appeared large, round, and coalesced into sheet-like formations with clear layers, indicating that mineralized nodules are late markers of osteogenic differentiation. Specifically, the CGRP-treated groups (CGRP-ADSCs and CGRP-ADSCs-CaAlg) showed a significantly higher number of calcium nodules at day 21 compared to the non-CGRP-treated groups (ADSCs and ADSCs-CaAlg, referred to as the basal induction groups), with statistical significance (0.0169 ± 0.0007 vs. 0.0083 ± 0.0011 and 0.0252 ± 0.0030 vs. 0.0166 ± 0.0006, both *P* < 0.05). This indicates that CGRP enhances the osteogenic differentiation of ADSCs. Among all four groups, the CGRP-ADSCs-CaAlg group exhibited the most prominent calcium nodule formation, demonstrating a synergistic effect between CGRP and calcium alginate gel in promoting the osteogenic differentiation of ADSCs.

### 3.7 RT-PCR detection of osteogenic gene expression

**FIGURE 7:**
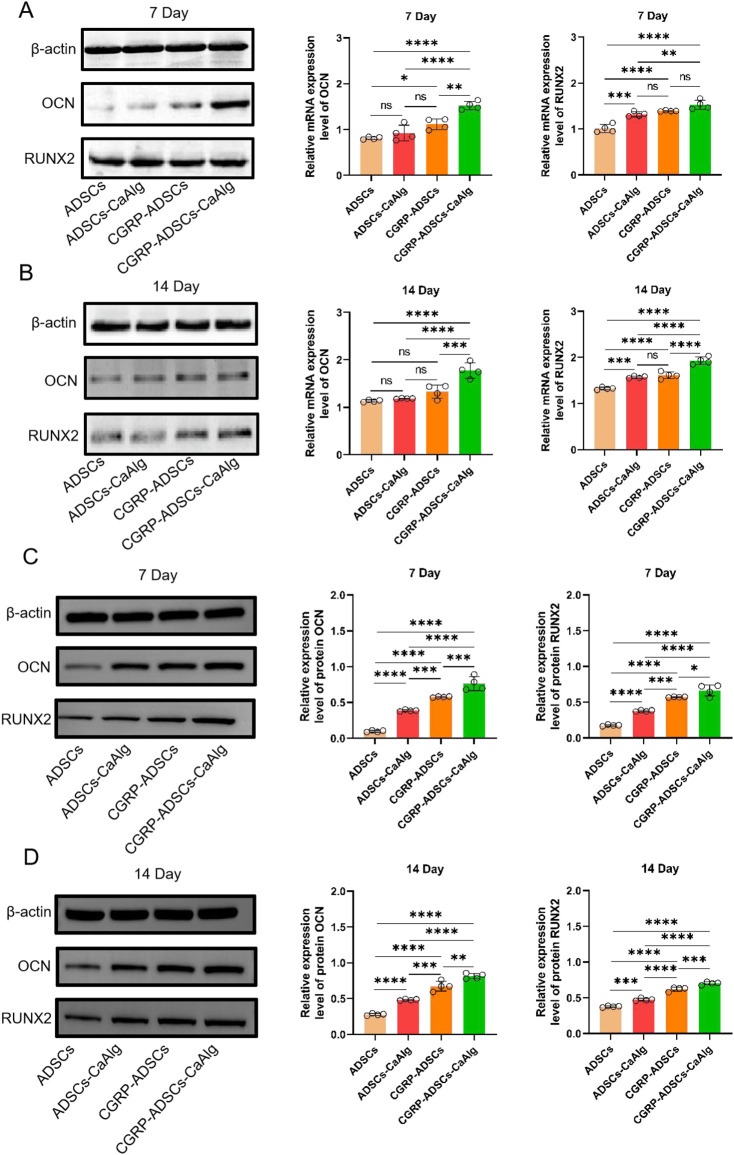
Expression of OCN and RUNX2 among all groups was detected by RT-PCR and Western blot. **(A,B)** OCN and RUNX2 mRNA expression and quantitative analysis of ADSCs in osteogenic induction culture among all groups after 7 days and 14 days by RT-PCR detection. The mRNA expression levels of OCN and RUNX2 increased over time in all groups at days 7 and 14. CGRP-ADSCs-CaAlg group exhibited significantly higher OCN mRNA expression on both day 7 and day 14 compared to the other three groups. Data are presented as mean ± SD (n = 4 biologically independent experiments). *P*-values were calculated using one-way ANOVA with Tukey’s *post hoc* test: ns means not significant. *P < 0.05, **P < 0.01, ***P < 0.001 and ****P < 0.0001. **(C,D)** OCN and RUNX2 protein expression and quantitative analysis of ADSCs in osteogenic induction culture among all groups after 7 days and 14 days by Western blot detection. The protein expression levels of OCN and RUNX2 increased over time in all groups at days 7 and 14. CGRP-ADSCs-CaAlg group exhibited significantly higher OCN and RUNX2 protein expression on both day 7 and day 14 compared to the other three groups. Data are presented as mean ± SD (n = 4 biologically independent experiments). *P*-values were calculated using one-way ANOVA with Tukey’s *post hoc* test: ns means not significant. *P < 0.05, **P < 0.01, ***P < 0.001 and ****P < 0.0001.

As shown in Figure 7, the mRNA expression levels of OCN and RUNX2 increased over time in all groups at days 7 and 14. Specifically, the CGRP-ADSCs-CaAlg group exhibited significantly higher OCN mRNA expression on both day 7 and day 14 compared to the other three groups (1.523 ± 0.091 vs. 0.812 ± 0.028, 0.923 ± 0.171, 1.116 ± 0.119 and 1.775 ± 0.163 vs. 1.132 ± 0.023, 1.184 ± 0.007, 1.329 ± 0.142, all *P* < 0.01). On day 7, the CGRP-ADSCs-CaAlg group also showed higher RUNX2 mRNA expression compared to the other three groups; however, this difference was not statistically significant when compared to the CGRP-ADSCs group (1.528 ± 0.101 vs. 1.392 ± 0.012, *P* = 0.090), but it was significant when compared to the ADSCs and ADSCs-CaAlg groups (1.528 ± 0.101 vs. 1.011 ± 0.090, 1.321 ± 0.053, both *P* < 0.01). By day 14, the CGRP-ADSCs-CaAlg group demonstrated significantly higher RUNX2 mRNA expression than the other three groups (1.928 ± 0.081 vs. 1.327 ± 0.027, 1.573 ± 0.025, 1.612 ± 0.073, all *P* < 0.001).

### 3.8 Western blot detection of osteogenic protein expression

As shown in Figure 7, the protein expression levels of OCN and RUNX2 increased over time in all groups at days 7 and 14. Specifically, the CGRP-ADSCs-CaAlg group exhibited significantly higher OCN protein expression on both day 7 and day 14 compared to the other three groups (0.764 ± 0.098 vs. 0.098 ± 0.013, 0.387 ± 0.011, 0.578 ± 0.008 and 0.813 ± 0.038 vs. 0.278 ± 0.014, 0.483 ± 0.010, 0.674 ± 0.069, all *P* < 0.001). Additionally, the CGRP-ADSCs-CaAlg group showed significantly higher RUNX2 protein expression on both day 7 and day 14 compared to the other three groups (0.664 ± 0.079 vs. 0.176 ± 0.010, 0.378 ± 0.010, 0.571 ± 0.006 and 0.702 ± 0.021 vs. 0.378 ± 0.010, 0.475 ± 0.019, 0.621 ± 0.029, all *P* < 0.05).

### 3.9 *In Vivo* osteogenic differentiation of ADSCs

**FIGURE 8:**
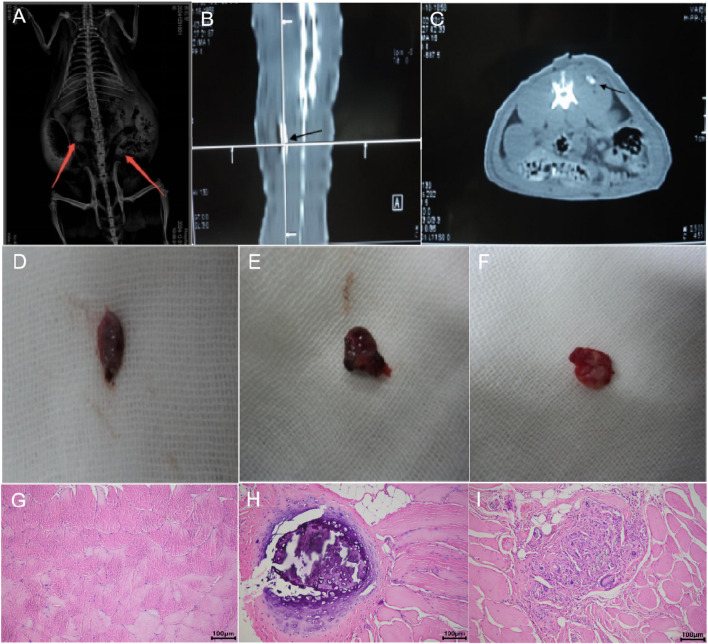
*In vivo* osteogenic evaluation. **(A)** X-ray imaging; **(B)** CT coronal view; **(C)** CT transverse view; **(D)** Gross morphology of the CaAlg group; **(E)** Gross morphology of the ADSCs-CaAlg group; **(F)** Gross morphology of the CGRP-ADSCs-CaAlg group; **(G)** H&E staining of the CaAlg group tissue (×200); **(H)** H&E staining of the ADSCs-CaAlg group tissue (×200); **(I)** H&E staining of the CGRP-ADSCs-CaAlg group tissue (×200). Results indicate that CGRP promotes the osteogenic differentiation of ADSCs loaded with CaAlg. The arrows indicate bone tissue. Scale bar = 100 µm.

Radiographic examination revealed high-density shadows in the left side (CGRP-ADSCs-CaAlg group) and lower right quadrant (ADSCs-CaAlg group) of SD rats on X-ray imaging, whereas no such shadows were observed in the upper right quadrant (CaAlg group) (Figure 8A). Further confirmation with CT scanning identified an oval-shaped ossicle measuring approximately 1.0 cm × 1.0 cm × 0.3 cm within the left dorsal muscle (Figures 8B,C), while no bone formation was detected in the control site (upper right quadrant). Gross observation at 12 weeks post-operation showed no new bone formation in the CaAlg control group; the implanted material had been absorbed and was surrounded by a thin layer of fibrous and muscular tissue (Figure 8D). In contrast, partial absorption of the scaffold was observed in the ADSCs-CaAlg group, with areas replaced by dense white newly formed bone (Figure 8E). The CGRP-ADSCs-CaAlg group exhibited complete encapsulation of the material by firm, well-defined white bone tissue (Figure 8F). Histological analysis via H&E staining further confirmed these findings. In the control group, only muscle and fibrous tissue were present, with no evidence of bone formation (Figure 8G). The ADSCs-CaAlg group demonstrated partial scaffold degradation, along with capillary in growth throughout the implant and the presence of osteoblasts and nascent bone tissue (Figure 8H). Notably, the CGRP-ADSCs-CaAlg group displayed mature woven bone and trabecular structures, with osteoblasts migrating into lacunae and aligned at the interface with newly formed bone. Surrounding the bone matrix were newly formed capillaries and mature connective tissue, while the scaffold material had largely degraded (Figure 8I), indicating successful *in vivo* osteogenic differentiation and integration.

## 4 Discussion

CGRP is a neuropeptide widely distributed in the peripheral and central nervous systems, with diverse biological effects. While initially studied for its roles in cardiovascular and neurological regulation (Shinbara et al., 2013; Porseva et al., 2012), CGRP has recently gained increasing attention for its critical functions in bone repair and regeneration (Chen et al., 2010; Tian et al., 2013; Zhang et al., 2016). It is abundantly expressed in bone and related tissues—such as bone marrow, periosteum, synovium, and surrounding soft tissues—where it serves as a key mediator between the nervous system and skeletal repair (Wang et al., 2023). CGRP receptors are highly expressed in bone tissue, macrophages, osteoblasts, and vascular structures (Li et al., 2021; Wang et al., 2023). Research indicates that CGRP promotes the osteogenic differentiation of multiple stem cell types, including ADSCs, BMSCs, periosteum-derived stem cells, synovium-derived stem cells, periodontal ligament stem cells, and dental pulp stem cells (Li et al., 2021; Zhang et al., 2016; Mi et al., 2021; Jiang et al., 2022; Zhao et al., 2024). Mechanistically, CGRP enhances osteoblast differentiation and proliferation by binding to cell surface receptors and inhibiting apoptosis (Zhao et al., 2024), while also suppressing osteoclastogenesis (Ishizuka et al., 2005). Beyond direct effects on bone cells, CGRP modulates angiogenesis and the immune microenvironment during bone repair, contributing to a neuro-vascular-bone regulatory network (Wang et al., 2023; Gao et al., 2021). It activates the Wnt/β-catenin pathway to promote osteogenic differentiation in BMSCs and protects osteoblasts from apoptosis (Wu et al., 2023; Zhou et al., 2016). Additionally, CGRP downregulates RANKL expression, inhibits the NF-κB pathway, and reduces TRAP and histone K expression in osteoclasts, thereby suppressing bone resorption and promoting bone formation (He et al., 2016; Wang et al., 2010; Yoo et al., 2014). CGRP also enhances endothelial differentiation of BMSCs and angiogenesis via the PI3K-AKT pathway (Mi et al., 2021). In immune modulation, CGRP promotes polarization of M0 macrophages toward the M2 phenotype, creating a favorable environment for bone regeneration and remodeling (Niedermair et al., 2020; Loi et al., 2016). While M1 macrophages contribute to early and mid-stage bone formation, M2 macrophages play a more critical role in later stages, particularly in matrix mineralization (Kong et al., 2024; Pajarinen et al., 2019).

In recent years, bone repair research has increasingly focused on tissue engineering strategies using seed cells such as BMSCs, osteoblasts, and ADSCs. BMSCs have been the most widely studied due to their strong differentiation capacity and proven efficacy (Zuk et al., 2001; Fei et al., 2021). However, their clinical use is limited by donor site morbidity, limited availability, and infection risks. ADSCs exhibit similar multilineage potential and osteogenic capability to BMSCs, with comparable gene expression profiles (Tsuji et al., 2014). Moreover, ADSCs offer advantages in cell yield and proliferation rate (Li et al., 2020), making them a promising alternative for bone and cartilage tissue engineering. Current ADSC-based scaffold strategies include pure ADSC constructs, ADSCs combined with drugs or growth factors, and genetically modified ADSC scaffolds. Both animal and clinical studies have confirmed the potential of ADSCs in repairing bone damage and large defects (Li et al., 2012).

Hydrogels are biomaterial scaffolds that form 3D networks and exhibit clinically favorable properties, including excellent biocompatibility, adhesiveness, degradability, and biomechanical stability (Karami et al., 2023; He et al., 2022). Among them, CaAlg stands out compared to conventional options such as synthetic polyethylene glycol (with low bioactivity), animal-derived gelatin (potentially allergenic), and chitosan (which requires harsh degradation conditions). Its innovation lies in a “natural safety” profile, combined with “rapid physical cross-linking” that enables injectability. This material also modulates the cellular microenvironment through “ion-controlled release and high water retention” and meets varied tissue repair needs thanks to its “broad mechanical tunability and ease of functionalization”. These attributes allow it to overcome typical limitations of traditional scaffolds—such as toxic residue, limited functionality, and poor adaptivity—offering a more viable pathway for clinical translation in tissue engineering. Indeed, CaAlg have been approved by the US Food and Drug Administration (FDA) and widely used in drug formulation, the food industry, wound dressings, as well as bone and cartilage tissue engineering (Zhao et al., 2009). Furthermore, its highly porous structure facilitates nutrient diffusion and cell-cell communication, while its plasticity enables adaptation to irregular defect geometries (Rana et al., 2014). Injectable CaAlg hydrogels have been demonstrated to support the osteogenic and chondrogenic differentiation of encapsulated mesenchymal stem cells (Iseki et al., 2024). The flexibility of alginate allows incorporation of bioactive factors and conformal filling of defects, while its 3D architecture promotes cell adhesion, proliferation, and nutrient exchange—making it a highly suitable scaffold for bone and cartilage regeneration.

In this study, ADSCs were isolated and cultured through a well-established protocol involving type I collagenase digestion, centrifugation, and adherence. Flow cytometry confirmed that the cells highly expressed mesenchymal stem cell markers—CD29 (98.63%), CD44 (97.99%), and CD105 (97.41%)—while showing minimal expression of hematopoietic markers CD31 (7.45%) and CD45 (0.53%). These results, supported by typical spindle-shaped morphology observed under microscopy, confirmed the mesenchymal identity of the ADSCs.When cultured within the CaAlg hydrogel scaffold, ADSCs exhibited robust proliferation over time, indicating favorable biocompatibility and cellular adaptation. According to CCK-8 assays, both CGRP and the CaAlg hydrogel promoted cell proliferation and differentiation, with CGRP playing the dominant role. This effect may be attributed to CGRP’s ability to induce osteogenic differentiation via specific signaling pathways, enhance angiogenic factor expression, and facilitate gap junctional intercellular communication (GJIC) (Ma et al., 2013; Bischoff et al., 2008).

ALP activity increased across all groups from day 3 to day 14, peaking at day 14 before declining. Significant differences were observed among groups at most time points (*P* < 0.01). The only exception was between the ADSCs and ADSCs-CaAlg groups on day 3 (*P* = 0.998); all other comparisons were statistically significant (*P* < 0.001). The 3D scaffold groups showed higher ALP activity than 2D cultures, and CGRP-treated groups exceeded basic induction groups. Osteogenic staining and quantification confirmed positive ALP and alizarin red staining on days 7 and 21. Although ALP expression decreased by day 21, mineralized nodule formation increased. The CGRP-ADSCs-CaAlg group exhibited the strongest early ALP activity and the most calcium deposition at later stages. At the molecular level, RT-PCR and Western blot analyses revealed that CGRP-induced groups had significantly elevated mRNA and protein levels of osteogenic markers RUNX2 and OCN at days 7 and 14 compared to basic induction groups. Furthermore, 3D cultures showed higher RUNX2 and OCN expression than 2D cultures. After 12 weeks of induction, *in vivo* osteogenesis was assessed. X-ray and micro-CT imaging detected localized high-density areas in both ADSCs-CaAlg and CGRP-ADSCs-CaAlg groups. Histological examination with H&E staining confirmed mature bone formation in these groups, while no bone formation was observed in the control group. The expression patterns of osteogenic markers ALP, RUNX2, and OCN reflected the differential osteogenic capacity of ADSCs across the experimental conditions.

## 5 Conclusion

In summary, CGRP and the CaAlg scaffold demonstrate a synergistic effect in promoting the osteogenic differentiation of ADSCs. CGRP can effectively induce ADSCs within a CaAlgl scaffold to form tissue-engineered bone *in vivo*. However, the current study did not investigate the underlying mechanisms of osteogenic differentiation. To date, the exact mechanism by which CGRP promotes osteogenesis in ADSCs remains unclear. However, emerging evidence suggests that CGRP may enhance osteogenic differentiation by activating the canonical Wnt/β-catenin signaling pathway while simultaneously inhibiting osteoblast apoptosis (Villa et al., 2003; Mrak et al., 2010). Future studies should focus on elucidating these molecular mechanisms and validating the signaling pathways involved in CGRP-mediated osteogenesis. At the same time, there is no comparative study with other scaffold materials. In future studies, comparative studies with other hydrogel scaffolds can be considered to highlight the unique advantages of this scaffold.

## Data Availability

The original contributions presented in the study are included in the article/supplementary material, further inquiries can be directed to the corresponding authors.
